# Maximizing anaerobic performance with repeated-sprint training in hypoxia: In search of an optimal altitude based on pulse oxygen saturation monitoring

**DOI:** 10.3389/fphys.2022.1010086

**Published:** 2022-10-12

**Authors:** Alexandre P. Gutknecht, Martin Gonzalez-Figueres, Thomas Brioche, Olivier Maurelli, Stéphane Perrey, François B. Favier

**Affiliations:** ^1^ DMEM, University of Montpellier, INRAE, Montpellier, France; ^2^ EuroMov Digital Health in Motion, University of Montpellier, IMT Mines Ales, Montpellier, France

**Keywords:** exercise, cycling, hypoxemia, heart rate variability, wingate test

## Abstract

**Purpose:** Repeated-sprint training in hypoxia (RSH) leads to great improvements in anaerobic performance. However, there is no consensus about the optimal level of hypoxia that should be used during training to maximize subsequent performances. This study aimed to establish whether such an optimal altitude can be determined and whether pulse oxygen saturation during RSH is correlated with training-induced improvement in performance.

**Methods:** Peak and mean power outputs of healthy young males [age (mean ± SD) 21.7 ± 1.4 years] were measured during a Wingate (30 s) and a repeated-sprint ability (RSA; 10 x 6-s sprint with 24-s recovery) test before and after RSH. Participants performed six cycling sessions comprising three sets of 8 x 6-s sprint with 24-s recovery in normobaric hypoxia at a simulated altitude of either 1,500 m, 2,100 m, or 3,200 m (*n* = 7 per group). Heart rate variability was assessed at rest and during recovery from Wingate test before and after RSH.

**Results:** The subjective rating of perceived exertion and the relative exercise intensity during training sessions did not differ between the three groups, contrary to pulse oxygen saturation (*p* < 0.001 between each group). Mean and peak power outputs were significantly increased in all groups after training, except for the mean power in the RSA test for the 3200 m group. Change in mean power on RSA test (+8.1 ± 6.6%) was the only performance parameter significantly correlated with pulse oxygen saturation during hypoxic training (*p* < 0.05, r = 0.44). The increase in LnRMSSD during recovery from the Wingate test was enhanced after training in the 1,500 m (+22%) but not in the two other groups (≈– 6%). Moreover, the increase in resting heart rate with standing after training was negatively correlated with SpO2 (*p* < 0.01, r =–0.63) suggesting that hypoxemia level during training differentially altered autonomic nervous system activity.

**Conclusion:** These data indicate that RSH performed as early as 1,500 m of altitude is effective in improving anaerobic performance in moderately trained subjects without strong association with pulse oxygen saturation monitoring during training.

## Introduction

The ability to repeat intense efforts with incomplete recovery (called RSA for Repeated-Sprint Ability) is an important physical component of performance in a broad spectrum of disciplines such as team and racket sports. The RSA relies on many factors, including metabolic (e.g., maximal oxygen uptake, H^+^ buffering) and neuromechanical factors (Girard et al., 2011). Accordingly, repeated-sprint training can be used to improve several physical abilities of athletes (Taylor et al., 2015). Faiss and others were the first to propose coupling RSA training with hypoxia exposure to further enhance repeated-sprint performance (Faiss et al., 2013). Furthermore, two reviews concluded that repeated-sprint training in hypoxia (RSH) induces greater improvement in mean repeated-sprint performance compared to the same training in normoxia for a wide range of sports including team sports, endurance sports, racket sports or combat sports (Brocherie et al., 2017; Millet et al., 2019). Interestingly, short term RSH benefits (i.e., increased mean and maximal repeated power output) have been observed in world-level rugby union players after only four sessions (Beard et al., 2019). One advantage of coupling repeated-sprint training with hypoxia is that it does not affect mechanical work or power sustained by the athletes during the training sessions contrary to the completion of aerobic training in altitude (Levine and Stray-Gundersen 1992), at least for altitudes below 3,500 m (Faiss et al., 2013; Brocherie et al., 2016; Tong et al., 2021; Townsend et al., 2021). Therefore, RSH protocol ensures keeping training intensity thereby maximizing the training-derived improvement in muscle qualities.

The underlying mechanisms that can contribute for improving repeated-sprint performance following RSH include an enhanced tolerance of metabolic acidosis and a higher glycolysis rate. Indeed, RSH increases expression of carbonic anhydrase III which is involved in buffering capacity, and addition of ambient hypoxia during sprint interval training or RSH further enhances activity of muscle glycolytic enzymes (Faiss et al., 2013; Puype et al., 2013). Moreover, RSH has been proposed to enhance muscle blood perfusion (Faiss et al., 2013) as well as the rate speed of resynthesis of phosphocreatine (Faiss et al., 2013; Brocherie et al., 2018). In fact, both glycolysis and muscle blood flow are regulated by the autonomic nervous system (Nonogaki 2000; Fisher and Paton 2012) whose activity is altered by hypoxia exposure (Zuzewicz et al., 1999; Barak et al., 2001). Noninvasive assessment of the autonomic nervous system activity can be performed through analysis of the heart rate variability (HRV), which is a reflection of the balance between sympathetic and parasympathetic tone (Task Force 1996). The link between aerobic performance and HRV, especially parasympathetic activity, is well documented (Melanson and Freedson 2001; Garet et al., 2004; Nummela et al., 2010). However, the evidence of any relationship between the HRV and RSA or anaerobic performances is far less clear. Indeed, training-induced improvement of RSA has been associated with either an increase (Buchheit et al., 2012; de Oliveira et al., 2013), a decrease (Buchheit et al., 2012; Merati et al., 2015) or no changes (Brechbuhl et al., 2020) in parasympathetic activity (root mean square of successive differences (RMSSD) and high frequency (HF) power). Acute hypoxia alters HRV (Wille et al., 2012; Krejčí et al., 2018) and may combine with repeated-sprint training to further stress autonomic nervous system. To the authors’ knowledge, only one study monitored resting HRV indices before and after RSH but failed to detect an association between performance enhancement and changes in HRV (Brechbuhl et al., 2020). However, the authors did not record HRV during recovery, especially parasympathetic reactivation characterizing fitness level (Buchheit 2014) which was found to increase after repeated-sprint training and high-intensity intermittent training in normoxia (Buchheit et al., 2008; Vernillo et al., 2015).

Most studies on RSH used normobaric hypoxia equivalent to about 3,000 m above sea level (Brocherie et al., 2017). Nevertheless, the level of simulated altitude required to promote the greatest gains in performance is still an unresolved issue and no study has compared the effects of RSH at different altitudes yet. Ensuring absolute intensity is maintained is important to identify the proper role of hypoxia level in the physiological mechanisms induced by RSH, excluding altitudes greater than 3,500 m (Brocherie et al., 2016; Tong et al., 2021; Townsend et al., 2021). Furthermore, there is a large inter-individual variability in human response to hypoxia. Indeed, the same inspired oxygen fraction (FiO_2_) leads to distinct values of pulse oxygen saturation (SpO_2_), which determines the degree of hypoxemia; and this variability increases with altitude level (Woorons and Richalet 2021). SpO_2_ is acknowledged as a systemic marker of organism’s adaptation to hypoxic conditions. Thus, SpO_2_ was shown to be correlated with various parameters including blood levels of catecholamines (Rostrup 1998), vascular-tone modulators (Ali et al., 2012), or lipid peroxidation (Bailey et al., 2001), as well as cerebral blood flow (Willie et al., 2014), pulmonary artery pressure (Mishra et al., 2013), micturition (Verratti et al., 2016), or oculomotor reflexes (Cymerman et al., 2005). Therefore, monitoring SpO_2_ might be helpful in explaining why some athletes would respond less to RSH as observed with aerobic training (de Paula and Niebauer 2012).

To sum up, the present study aimed to determine whether anaerobic performance was differentially improved after RSH performed at three distinct altitude levels, and whether changes in performance were related to the SpO_2_ level achieved during training sessions. Furthermore, it was determined how the moderate-to high-altitude levels used for RSH alter HRV at rest and during recovery. It was hypothesized that the more participants desaturated during the RSH sessions, the more performance would be improved.

## Materials and methods

### Participants

Twenty-four male students from the Sports Sciences Faculty volunteered to take part in this study. Three subjects dropped out during the study because of injury unrelated to our protocol (*n* = 2) and restrictions associated with the coronavirus disease-2019 pandemic (*n* = 1). Thus, 21 participants were included in the study [age (mean ± SD) 21.7 ± 1.4 years; height 176.3 ± 7.8 cm; weight 68.5 ± 6.3 kg]. Inclusion criteria were 1) the absence of intolerance to moderate hypoxia, 2) no stay to an altitude above 1,500 m during the 2 months preceding the experiment, and 3) being accustomed to intense efforts such as repeated sprints. All participants signed a written informed consent before participating in the experimental protocol. The study was approved by the local Ethics Committee (IRB-EM 2002B, EuroMov-Montpellier) and was conducted in accordance with the Declaration of Helsinki.

### Overall experimental design

The participants were paired based on the initial performance obtained on the Wingate test and then randomly assigned to one of the following groups: 1,500 m (*n* = 7), 2,100 m (*n* = 7), and 3,200 m (*n* = 7) to balance the groups. The RSH sessions were conducted at a simulated altitude (normobaric hypoxia) corresponding to the dedicated group. These three altitudes were chosen for two main reasons. First, one of the initial goals of the study was to correlate participants' SpO_2_ data during RSH with subsequent changes in performance. In this sense, it seemed more relevant to obtain a continuum over a wide range of SpO_2_, and it was shown that the decrease in SpO_2_ during intense exercise worsened beyond 1,500 m (Woorons and Richalet 2021). Second, altitude above 3,500 m affects performance on repeated-sprint exercise (Brocherie et al., 2016; Tong et al., 2021; Townsend et al., 2021), and no study showed performance improvement after RSH above such altitude (Galvin et al., 2013; Camacho-Cardenosa et al., 2020). Training consisted of six sessions of RSH over a 2-week period on a friction-loaded cycle ergometer (Monark type 818E, Stockholm, Sweden) equipped with a strain gauge (interface MFG type, Scottsdale, AZ, United States). The participants performed a Wingate test and a RSA test before and after the training program. All testing sessions were carried out at 75 m in an air-conditioned room with ambient temperature maintained around 21°C.

### Training sessions

Training sessions consisted of a 7-min warm-up [resistance 2% of body mass (BM)] with two progressive accelerations of 10 s at the 5th and 6th min. Then, after 1 min of passive rest, the participants performed three sets of eight repetitions of 6-s maximal sprint interspersed with 24 s of passive recovery on the cycle ergometer. Resistance was initially set to 5% of BM for each session and could be slightly adjusted to ensure the best performance based on participant’s feeling. A LCD screen informed participants of the time count-down and the number of sprints.

Altitude exposure was simulated by a gas-mixing device (Altitrainer^®^, SMTEC S.A., Nyon, Switzerland). The participants started to breathe the nitrogen-enriched gas mixture *via* a facemask at the beginning of the warm-up. They were told that the simulated altitude was around 3,000 m, but they were blind for the hypoxia level to which they actually trained (1,500 m, 2,100 m, or 3,200 m corresponding to a FiO_2_ of 17.5, 16.2, or 14.1%, respectively). SpO_2_ was recorded with a pulse oximeter (pulsox®-300i, Konica Minolta Inc., Osaka, Japan) throughout all the training sessions (including the warm-up) at a frequency of 1 Hz. The oximeter screen was hidden from participants as well as the experimenter. The participants were asked as often as needed not to clench the hand connected to the oximeter to avoid artefacts in SpO_2_ values. They were also asked to determine ratings of perceived exertion (RPE) on a 6–20 Borg’s scale after the warm-up and each of the three sets. For the first session, the participants were asked to manage the effort made to complete the three sets.

### Testing sessions and analysis

The pre- and post-tests took place in the morning or the afternoon, but at the same time of the day (±1 h) for each participant to avoid bias due to circadian variations of performance (Knaier et al., 2019). The composition of the two meals preceding the pre-tests were recorded and participants were asked to eat the same menus before the post-tests. Moreover, participants were asked not to consume caffeine or alcohol 24 h before each test. Post-tests were performed 3–4 (Wingate) and 5–6 days (RSA) after the last training session.

During all tests and training sessions, the participants were given strong verbal encouragement by the same experimenter. Mechanical power output (W) was recorded at a frequency of 1 Hz using PowerTap P1 pedals (CycleOps, Madisson, United States) connected to the PowerTap application on an Ipad Pro (Apple Inc., United States) (Pallarés and Lillo-Bevia 2018). The digital tablet was positioned behind the participants in such way the power output and heart rate could not be seen during the tests.

Each participant completed the self-administrated 65-item Profile of Mood States questionnaire during the warm-up preceding every test. Mood states were interpreted through seven domains: anger, anxiety, confusion, depression, fatigue, friendliness, and vigor (Gueldich et al., 2019).

HRV was measured at rest before the RSA test and during the early recovery of the Wingate test. Measurements were conducted in a quiet room with participants wearing a Polar H10 sensor (Polar Electro, Kempele, Finland) connected to the validated Elite HRV Application (Elite HRV LLC, Asheville, North Caroline, United States) (Perrotta et al., 2017). Raw data were analyzed with Kubios HRV analysis software (Kubios HRV Analysis, Biosignal Analysis and Medical Imaging Group, Kuopio, Finland) with a medium threshold of correction. Before the RSA test, participants sat on the ground with legs stretched out for 8 min and stood up for 7 min. HRV indices were calculated from the last stationary 4 min for each position and during the 6 min of recovery immediately after the completion of the Wingate test with the participants sitting with legs horizontally stretched out. In the time-domain, RMSSD as an index of the parasympathetic modulation (Task Force 1996) was calculated. In the frequency-domain, the following HRV indices were calculated: low-frequency spectral power (LF, 0.04–0.15 Hz), which reflects predominantly the sympathetic system influence, and HF spectral power (HF, 0.15–0.4 Hz), which reflects the parasympathetic (vagal) activity (Task Force 1996).

### Wingate test

The warm-up prior to the Wingate test consisted of 6 min at a self-selected pedaling rate with a resistance set to 2% BM. After 2 min of passive recovery, the participants were asked to develop the highest mechanical power through the 30 s of the test with a resistance of 5% BM while remaining seated on the saddle. Peak and mean powers characterize respectively anaerobic power and capacity (de Poli et al., 2021). Blood lactate was measured from the fingertip (Lactate scout+, EKF Diagnostics, Germany) at rest, at 5 and 6 min after exercise cessation. The highest value between samples from the 5th and 6th min was used for statistical analysis. Another blood sample was taken at rest to determine hemoglobin concentration (Hemo-Control, EKF Diagnostics). Data from one participant of the 1,500 m group were not analyzed because the recording of power output failed during the Wingate test.

### Repeated-sprint ability test

The participants carried out the RSA test 48 h apart from the Wingate test. The warm-up was composed of 5 min at a self-selected pedaling rate with a resistance set to 2% BM with two 4-s maximal sprints (resistance 5% BM) at the 3rd and 5th min. After 2 min of recovery, the participants performed 10 maximal sprints of 6 s (resistance 5% BM) interspersed with 24 s of passive rest. The participants were encouraged to reach the highest mechanical power on every sprint and were not aware of the total number of sprints to avoid pacing strategy. The average power output over the entire test (mean power) and the average of peak powers over each of the 10 sprints (peak power) were computed for the RSA test. Blood lactate was measured at rest, at 2nd and 3rd min after the 10th sprint. The highest value between samples from the 2nd and 3rd min was used for statistical analysis.

### Statistical analysis

Values are presented as means ± standard deviations. We used the Shapiro–Wilk test to examine the normal distribution of the variables. Since HRV indices were not normally distributed, we used natural logarithm (Ln) transformation to obtain a normal distribution and allow parametric statistical comparisons. Mechanical power, profile of mood states, lactate, and hemoglobin concentration data were analyzed using a two-way repeated-measures analysis of variance (ANOVA) with time (pre- vs. post-training) as within-subject factor and hypoxia level (3 groups: 1,500 m, 2,100 m, and 3,200 m) as between-subject factor. Mechanical power data of RSA tests were also analyzed with a three-way repeated-measures ANOVA with time (pre- vs. post- training) and sprint number (from 1 to 10) as within-subject factors and hypoxia level as between-subject factor. RPE data were analyzed with a two-way repeated-measures ANOVA with time (warm-up vs. set 1 vs. set 2 vs. set 3) as within-subject factor, and hypoxia level as between-subject factor. The sphericity and the equality of variance assumptions were tested with Mauchly’s and Levene’s tests, respectively. The Greenhouse-Geisser correction was used when sphericity was not met. Tukey’s post hoc was used when ANOVA was significant. Pearson correlation was used to determine the association between changes in performance and mean SpO_2_ during RSH. The level of significance was set to 0.05 for all tests. Effect sizes (ES) for the ANOVA were calculated using partial eta squared (η_p_^2^) for group, time, and interaction of these two factors. ES was considered small when 0.01 < η_p_^2^ ≤ 0.06, medium when 0.06 < η_p_^2^ ≤ 0.14, and when large η_p_^2^ > 0.14. All statistical analyses were performed using the Jamovi Project software (version 1.0.7.0).

## Results

### Training parameters

**FIGURE 1:**
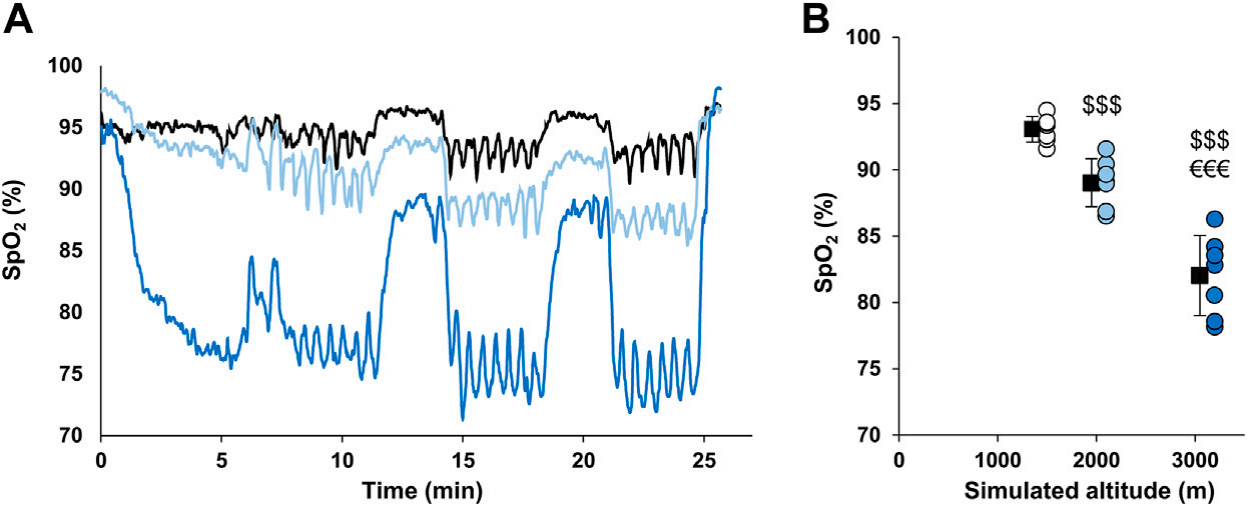
SpO_2_ values during hypoxic training sessions. **(A)** Time course of SpO_2_ during RSH sessions in representative participants from each group. Participants from 1,500 m, 2,100 m, and 3,200 m groups are represented by black, light blue and dark blue lines, respectively. **(B)** Mean SpO_2_ during the whole RSH sessions. Individual and mean group values are represented by circles and squares, respectively. ^$$$^ *p* < 0.001 vs. 1,500 m group; ^€€€^ *p* < 0.001 vs. 2,100 m group.

The time course of SpO_2_ during RSH sessions of a representative subject from each group is presented in Figure 1A. Mean SpO_2_ during all training sessions was 93.1 ± 0.96, 89.1 ± 1.82, and 82 ± 3.03% for the 1,500 m, 2,100 m, and 3,200 m groups, respectively (all significantly different, *p* < 0.001; Figure 1B). The evolution of the mean SpO_2_ between the first two and the last two sessions was different between groups (time × group interaction, *p* = 0.03) with the 3,200 m group displaying opposite variation (decrease of 1.1% in SpO_2_) as compared to the two other groups (increase of 0.3 and 0.5% in SpO_2_).

The mean power output over the whole sessions was 8.47 ± 0.45, 8.92 ± 0.7, and 9.45 ± 0.58 W/kg for the 1,500 m, 2,100 m, and 3,200 m groups, respectively with the 3,200 m group having significantly greater value compared to the 1,500 m group (*p* = 0.02). However, when normalizing this power output by the mean power output sustained during the pre-RSA test, no significant differences were observed between groups (88.1 ± 5.5, 90.8 ± 4.3, and 86.4 ± 3.1%; *p* = 0.19). The RPE increased significantly along the sessions but was not different between groups (Table 1).

**TABLE 1:** Ratings of perceived exertion during the training sessions.

	1,500 m	2,100 m	3,200 m	ANOVA p values (η_p_^2^)		
				Group	Time	G x T
RPE						
Warm-up^a^	8.89 ± 1.75	10.69 ± 1.26	10.14 ± 1.78			
Set 1^b^	13.72 ± 0.93	15.33 ± 1.49	14.45 ± 1.91	0.3	**<0.001**	0.53
Set 2^c^	16.40 ± 0.93	16.96 ± 1.30	16.54 ± 2.12	(0.12)	**(0.92)**	(0.08)
Set 3^d^	17.85 ± 1.20	18.06 ± 1.46	17.93 ± 1.72			

G x T: group × time interaction. Time points with different superscript letters are significantly different according to Tukey’s post hoc test (*p* < 0.05). *p*-values and effect size are bold when significant and of large magnitude, respectively.

### Performance on the wingate and repeated-sprint ability tests

**FIGURE 2:**
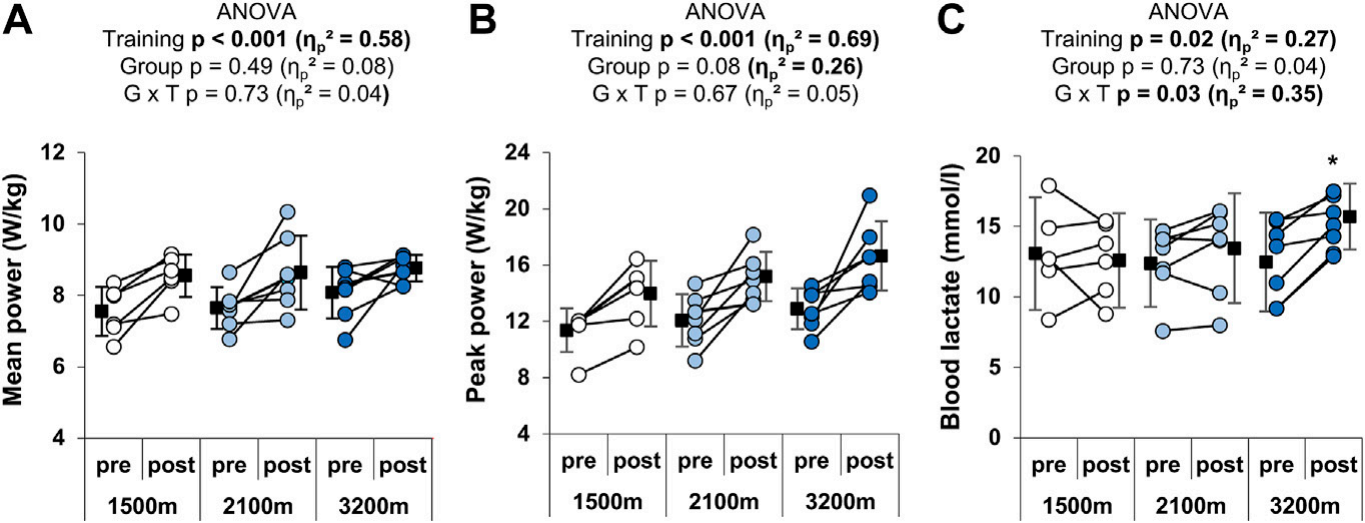
Individual and mean performances during the Wingate test. **(A)** Mean and **(B)** peak power outputs in W/kg during the 30 s, and **(C)** peak blood lactate after the test. *p < 0.05 vs. pre-training. Circles and squares represent individual and mean data, respectively. *p*-values and effects size are bold when significant and of large magnitude, respectively. G x T: group × time interaction.

RSH training significantly increased mean (+11.9 ± 11.1%; η_p_^2^ = 0.58) and peak (+27 ± 19.4%; η_p_^2^ = 0.69) power outputs during the Wingate test, but hypoxia level did not alter the gain of peak and mean power output (no group × time interaction; Figures 2A,B). Maximal blood lactate after the Wingate test was also increased following RSH training (+10.1 ± 18.2%), and this effect was greater in the 3,200 m group (+23 ± 16.9%; significant group × time interaction; Figure 2C).

**FIGURE 3:**
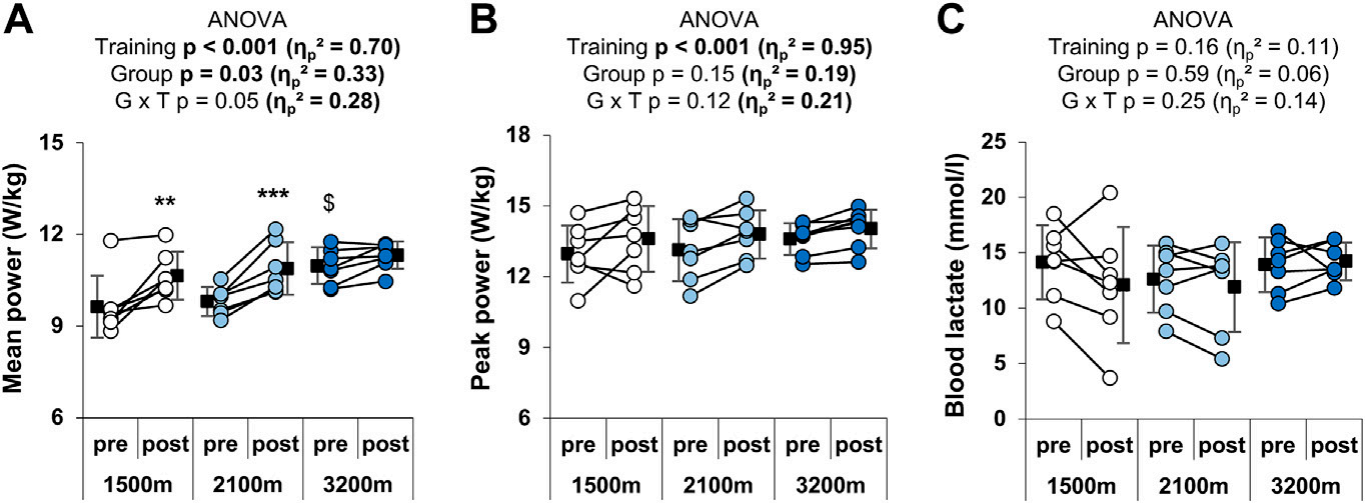
Individual and mean performances during the RSA test. **(A)** Mean power and **(B)** mean peak power outputs in W/kg over the 10 sprints, and **(C)** peak blood lactate after the test. **p < 0.01 and ***p < 0.001 vs. pre-training; $*p* < 0.05 vs. 1,500 m. Circles and squares represent individual and mean data, respectively. *p*-values and effects size are bold when significant and of large magnitude, respectively. G x T: group × time interaction.

RSH training had a main positive effect on both the mean (+8.1 ± 6.6%; η_p_^2^ = 0.70) and the peak power output (+4.6 ± 6.2%; η_p_^2^ = 0.95) over the RSA test (Figures 3A,B). The effect of group × time interaction was large (η_p_^2^ = 0.28) and approached significance (*p* = 0.05) for the mean power output. Post-hoc analyses revealed that mean power output was significantly increased in the 1,500 m (+10.6 ± 8.1%) and the 2,100 m groups (+10.5 ± 4.9%), but not in the 3,200 m group (+3.1 ± 3.4%). Furthermore, the pre-test value of the 3,200 m group was higher than the pre-test value of the 1,500 m group (*p* = 0.02). The three-way ANOVA gave no further information for the peak power output (significant main effects of time and sprint number, but no significant group x time or group x sprint number × time interaction) or the mean power output (significant main effects of group, time and sprint number; group x time *p* = 0.05; no significant group x sprint number × time interaction). Before training, participants from the 1,500 m, the 2,100 m, and the 3,200 m groups respectively performed 4.9 ± 2.3, 5.9 ± 3, and 4.4 ± 2.1 sprints with a power output greater than 85% of the best mean power output produced during the test versus 4.6 ± 3.6., 4.6 ± 3, and 5.6 ± 2.6 after training. There was no significant effect of either group (*p* = 0.92; η_p_^2^ = 0.01), time (*p* = 0.84; η_p_^2^ < 0.01), or interaction of these factors (*p* = 0.40; η_p_^2^ = 0.10). Any effect of time or altitude level was observed on blood lactate after the RSA test (Figure 3C).

**FIGURE 4:**
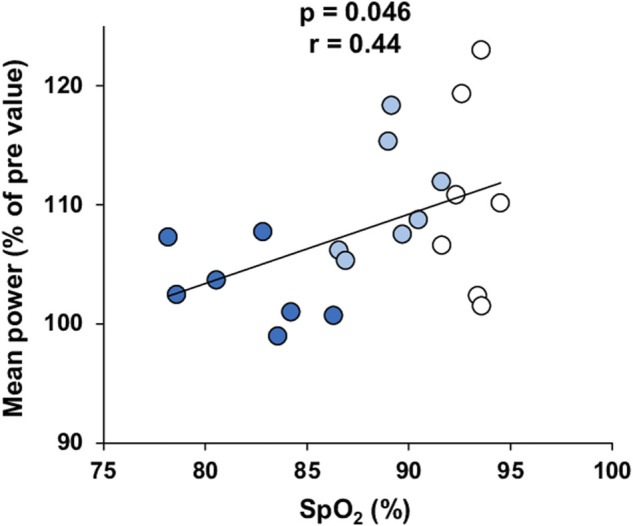
Correlation between the changes in mean power output on the RSA test after training and mean SpO_2_ measured during RSH. Participants of the 1,500 m, 2,100 m, and 3,200 m groups are represented by white, light blue and dark blue dots, respectively.

Changes in performance outputs on the RSA and the Wingate tests were plotted against the mean SpO_2_ during the RSH sessions. SpO_2_ was not correlated with changes in peak or mean power on the Wingate test. In contrast, changes in mean (but not peak) power on the RSA test were significantly correlated with SpO_2_ (*p* = 0.046, r = 0.44; Figure 4). The changes in performances were not related to the evolution of SpO_2_ from the start (mean of the first two sessions) and the end of the training (last two sessions).

### Heart rate variability indices

**FIGURE 5:**
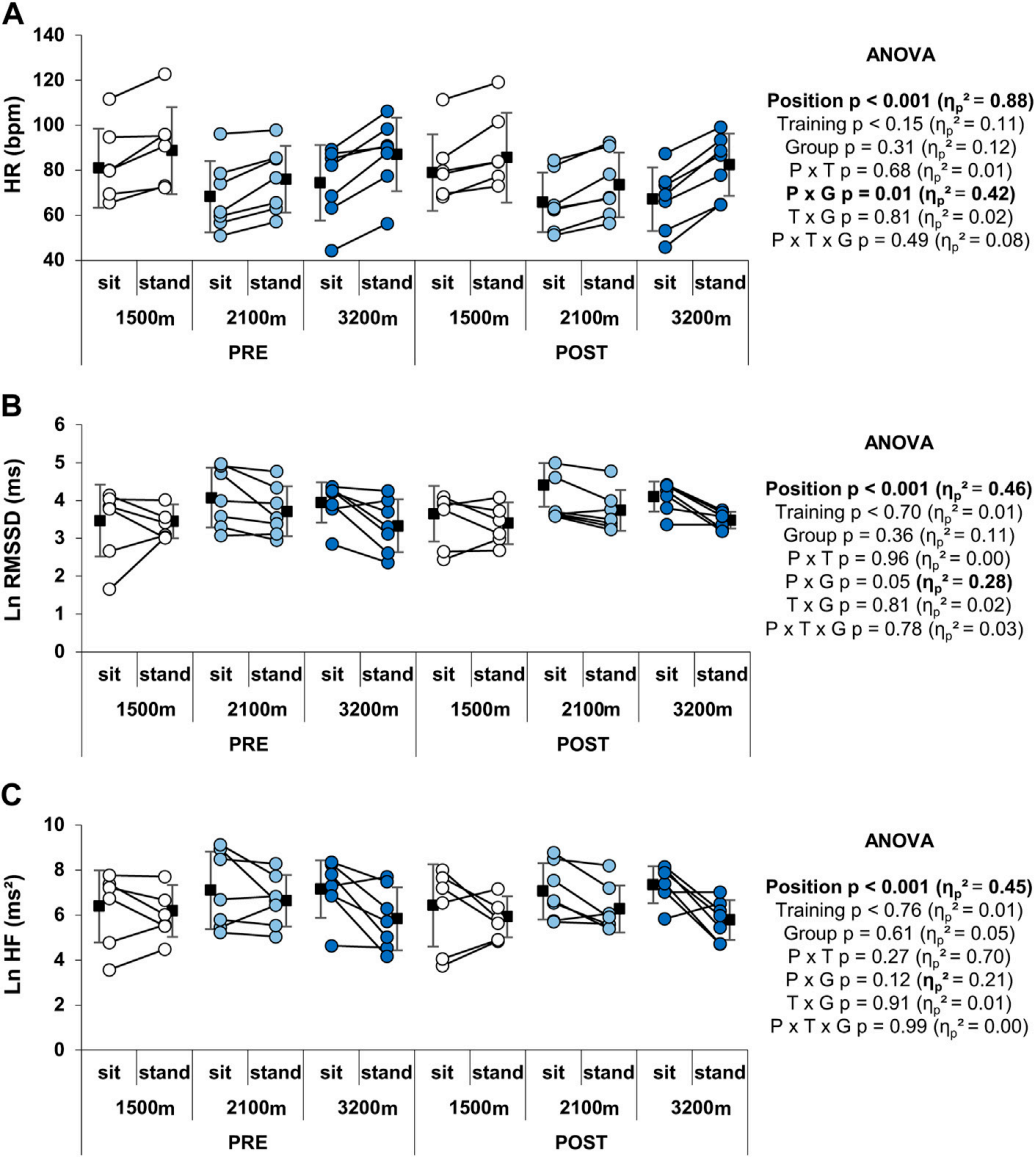
Individual and mean HR and HRV indices at rest. **(A)** HR, **(B)** LnRMSSD, and **(C)** LnHF values. Circles and squares represent individual and mean data, respectively. *p*-values and effects size are bold when significant and of large magnitude, respectively. P x G: position × group interaction; P x T: position × time interaction; P x T x G: position x time × group interaction; T x G: time × group interaction.

RSH did not alter resting HR in either position (sitting or standing), while standing had a main positive effect on HR (*p* < 0.001, Figure 5A). There was no main effect of group but a significant group × position interaction on HR (*p* = 0.01). Indeed, the main increase in HR with standing was 7.1 ± 5.1 and 7.6 ± 3.7 bpm for the 1,500 m and 2,100 m groups respectively, whereas it reached 13.8 ± 5.1 bpm for the 3,200 m group. LnRMSSD and LnHF were significantly reduced in the upright position compared to sitting (*p* < 0.001, large ES), whereas RSH training and altitude level had no main effects on these parameters (Figures 5B,C). The group × position interaction on LnRMSSD (large ES) was significant (*p* = 0.05), with the 3,200 m but not the 1,500 m and the 2,100 m group displaying a significant reduction with the standing position, independently from the RSH effect. No significant effects were observed either on LnLF or Ln(LF + HF) (not shown).

Neither time nor altitude level (group) had any significant effect on HRV indices (LnRMSSD, LnHF, LnLF, Ln(LF + HF)) during recovery from the Wingate test (Table 2). Time × group interaction was significant for LnRMSSD (*p* = 0.03), LnLF (*p* = 0.02) and Ln(LF + HF) (*p* = 0.02) with 1,500 m group exhibiting an increase in these indices after training (+22 ± 10, +18 ± 1, and +16 ± 1%, respectively), while 2,100 m and 3,200 m groups showing opposite variations (–8 ± 0.1, –12 ± 2, and –12 ± 4% for the 2,100 m group, –5 ± 16, –7 ± 12, and –6 ± 14% for the 3,200 m group). HR recovery at 1 and 6 min was not altered by time or hypoxia level (Table 2).

**TABLE 2:** HR and HRV indices during recovery from the Wingate test.

	1,500 m		2,100 m		3,200 m		ANOVA p values (η_p_^2^)		
	Pre	Post	Pre	Post	Pre	Post	Group	Time	G x T
HRR 1 min (%)	21.90 ± 6.58	21.83 ± 6.73	25.02 ± 6.91	22.55 ± 7.27	25.46 ± 4.61	25.05 ± 4.82	0.64 (0.06)	0.35 (0.06)	0.59 (0.07)
HRR 6 min (%)	45.91 ± 8.39	48.79 ± 6.51	51.19 ± 9.73	49.21 ± 9.91	48.22 ± 8.85	46.43 ± 6.04	0.79 (0.03)	0.79 (0.01)	0.18 **(0.22)**
Ln RMSSD (ms)	1.67 ± 0.49	2.04 ± 0.32	2.57 ± 0.85	2.38 ± 0.84	1.98 ± 0.90	1.89 ± 0.58	0.29 **(0.16)**	0.74 (0.01)	**0.03 (0.38)**
Ln HF (ms^2^)	2.20 ± 0.81	2.49 ± 0.96	4.44 ± 1.62	3.43 ± 2.08	2.96 ± 1.86	2.79 ± 0.90	0.19 **(0.21)**	0.26 (0.09)	0.15 **(0.24)**
Ln LF (ms^2^)	3.97 ± 0.93	4.68 ± 0.98	5.20 ± 1.45	4.59 ± 1.58	4.27 ± 1.53	3.98 ± 1.01	0.56 (0.08)	0.71 (0.01)	**0.02 (0.44)**
Ln (LF + HF) (ms^2^)	4.14 ± 0.90	4.81 ± 0.94	5.62 ± 1.46	4.93 ± 1.69	4.55 ± 1.57	4.27 ± 0.94	0.45 (0.11)	0.57 (0.02)	**0.02 (0.42)**

G x T: group × time interaction; HF, high-frequency spectral power; HRR, heart rate recovery after 1 and 6 min of rest in percentage of initial heart rate; LF, low-frequency spectral power; Ln, natural logarithm; RMSSD: root mean square differences of successive heart beat intervals. *p*-values and effect size are bold when significant and of large magnitude, respectively.

Analysis of the profile of mood state showed an improvement of the overall mood state (decrease in the total score) before starting the Wingate test after the RSH training (Table 3). This effect was associated with specific improvement in anxiety, confusion, depression, and friendliness, but not in fatigue or vigor subscales (not shown). The main effect of time on all these scores was not different between groups (no group × time interaction). Regarding both the total or subscales scores, mood state was not altered between pre- and post-tests for RSA (Table 3 and data not shown). Hemoglobin concentration was not affected by either training or hypoxia level (Table 3).

**TABLE 3:** POMS total score and hemoglobin concentration.

	1,500 m		2,100 m		3,200 m		ANOVA p values (η_p_^2^)		
	Pre	Post	Pre	Post	Pre	Post	Group	Time	G x T
POMS									
Wingate	48.57 ± 20.18	37.14 ± 18.86	42.00 ± 11.39	38.71 ± 13.95	54.71 ± 24.23	42.86 ± 26.60	0.69 (0.04)	**0.016 (0.28)**	0.51 (0.07)
RSA	37.71 ± 20.96	33.14 ± 18.42	41.83 ± 10.52	41.17 ± 17.79	41.14 ± 19.75	41.57 ± 24.08	0.78 (0.03)	0.58 (0.02)	0.75 (0.03)
[Hb] (g/dl)	14.93 ± 1.37	15.05 ± 1.67	14.33 ± 1.23	15.24 ± 1.48	14.26 ± 1.13	13.97 ± 1.15	0.29 (0.13)	0.51 (0.03)	0.43 (0.10)

G x T: group × time interaction; Hb, hemoglobin; POMS, profile of mood state. *p*-values and effect size are bold when significant and of large magnitude, respectively.

## Discussion

The present study is the first to compare the effects of RSH at three altitude levels (below 3,500 m) on anaerobic performance. The results showed that six sessions of RSH are effective for improving anaerobic power and capacity assessed through the Wingate and the RSA tests in moderately trained young males. Moreover, the highest altitude used (3,200 m) did not lead to further enhancement compared to 1,500 and 2,100 m, similar to the data of Warnier et al. (2020) who did not find differences in the effects of sprint interval training at either 2,000, 3,000, and 4,000 m. In accordance, there was no correlation between individual SpO_2_ during training and subsequent performance changes.

Previous findings on RSH support that hypoxia does not alter exercise intensity during repeated-sprints compared to normoxia, at least for FiO_2_ > 14% or below 3,400 m (Billaut et al., 2013; Faiss et al., 2013; Bowtell et al., 2014; Vasquez-Bonilla et al., 2021). The present study unveiled that there was no difference between the three groups concerning exercise intensity assessed by both subjective (RPE) and objective (mechanical power) variables. Thus, the potential differences in performance gain between groups would rely on hypoxia severity rather than a training load effect. Performance improvement of participants from the 3,200 m group on the RSA test (+3.1% for mean and peak power outputs) was slightly below that in published studies using RSH on cycle ergometer close to altitude of 3,000 m (about + 5–10% for both mean and peak power outputs) (Faiss et al., 2013; Goods et al., 2015; Beard et al., 2019). Although far less authors investigated effects of RSH with altitude around 2,000 m, it has been shown that RSH on cycle at 2,200 m induced a 10% increase in mean power on RSA and Wingate tests (Gatterer et al., 2018) which is consistent with the changes recorded in the 2,100 m group (+10.5 and 13.1% in mean power on the RSA and Wingate test, respectively).

Pulse oxygen saturation is a non-invasive and reliable proxy for determination of the physiological response during short hypoxic exposures (Woorons and Richalet 2021). Regarding studies on RSH, differences in experimental protocols (e.g., sprint duration, timing of SpO_2_ measurement, hypoxia delivered *via* mask or tent) may limit global comparisons of SpO_2_ values between studies (Billaut et al., 2013; Morrison et al., 2019; Vasquez-Bonilla et al., 2021). However, works with similar experimental conditions to the present one recorded values ranging from 82.6 to 85.6% for simulated altitudes around 3,000 m which is consistent with the value of the 3,200 m group (Billaut et al., 2013; Kasai et al., 2019; Yamaguchi et al., 2021). As expected, participants exhibited marked inter- and intra-group differences on SpO_2_ during RSH, with individual values ranging from 78.2 to 94.5%, allowing assessment of the relationship between hypoxemia and gains in performance. Hypothesis was made that marked hypoxemia will be associated with physiological adaptations triggered by hypoxia-inducible factor 1, which has been shown to be increased in blood and skeletal muscle after a single or several sessions of repeated-sprint exercise in hypoxia but not in normoxia (Faiss et al., 2013; Brocherie et al., 2018; Nava et al., 2022; Pramkratok et al., 2022). Indeed, hypoxia-inducible factor 1 is known to promote the transcription of glycolytic enzymes (reviewed in (Favier et al., 2015)). Although the greater increase in blood lactate after training in the 3,200 m group compared to the other two groups suggests a greater participation of glycolytic pathways, the present results did not show that improvements on the Wingate test depend upon the severity of hypoxemia experienced during training sessions. In contrast, there was a moderate positive correlation between the mean SpO_2_ during the RSH and the changes in mean power on the RSA test which requires non-negligible contribution of aerobic metabolism (Girard et al., 2011). According to authors’ knowledge, only one study reported the relationship between hypoxia-induced physiological adaptations and oxygen saturation during training. Indeed, it has been shown that improvements in exercise performance, maximal oxygen uptake and cardiac output in horses during an incremental test following 4 weeks of short high-intensity training at 15% FiO_2_ were correlated with the arterial oxygen saturation during hypoxic exercise (Mukai et al., 2020). The authors speculated that hypoxic training was too stressful for horses that developed the more severe hypoxemia leading to a probable overreaching state. Altogether, these data and results from the present study suggest that high hypoxemia during intense training under hypoxia could lead to lower improvement of performance on tests involving aerobic metabolism.

In the present protocol, HRV of the participants was recorded in attempt to observe whether hypoxia level modulated the stress induced by the RSH training. Indeed, acute hypoxia is known to alter autonomic nervous system activity with an increase in sympathetic-parasympathetic balance (e.g., increase in HR and blood lactate concentration, decrease in RMSSD or in HF power (Wille et al., 2012; Krejčí et al., 2018)). Intense exercise, such as repeated-sprints, and hypoxia may thus represent additive stimuli on autonomic nervous system activity. There were no striking changes in the indices of cardiac autonomic activity after training, consistently with a recent study (Brechbuhl et al., 2020). However, there was a moderate negative correlation between SpO_2_ during sessions and the increase in HR while standing after training (r = - 0.63; *p* = 0.003), suggesting that the level of hypoxemia during RSH may influence HRV. In addition, the 1,500 m group displayed distinct response to the Wingate test evidenced by significant time × group interaction on spectral power density of LF + HF and LnRMSSD after training compared to the two other groups. Although assessment of vagal tone (RMSSD) and global HRV (absolute power of LF + HF) after supramaximal exercise is less documented compared to aerobic exercise, an increase in these parameters could be a sign of a positive adaptation to training load (Buchheit et al., 2008; Vernillo et al., 2015). Altogether, these data are consistent with a greater training-induced stress in the participants displaying important desaturation, although participants of the 3,200 m group did not display more patterns of fatigue (not shown) that have been characterized in endurance trained subjects (Schmitt et al., 2018). It would have been interesting to schedule another post-test session later to see if the 3,200 m participants would have performed better than at 3–6 days after the last training session. Other parameters may have influenced the present results; for instance, chronic hypoxia is known to induce polycythemia (Fulco et al., 2000; Millet et al., 2010; de Paula and Niebauer 2012). However, it is unlikely that the hypoxic stimulus associated with RSH training is sufficient to induce significant changes in hemoglobin concentration (present results and (Faiss et al., 2013)), while others indicated higher hematocrit and hemoglobin concentration at 2 and 14 days after RSH intervention (Camacho-Cardenosa et al., 2017). The psychological state can also alter performance since the POMS score and Wingate-test performance are significantly related (Hill and Chtourou 2020). Inter-group differences in overall mood state or fatigue sub-scale before post-tests were not denoted, suggesting that hypoxic level had no major effect on these items. Some studies questioned the importance of blind design in RSH studies implying that a placebo effect may interfere with performance (Montero and Lundby 2017). However, the effective blinding of participants is sometimes difficult to set up when working with hypoxia. In the present study, the participants knew that training sessions were performed under hypoxic conditions but were naive about the exact altitude level. The participants’ beliefs might thus have contributed to the increase in performance between pre- and post-tests irrespective of the altitude level. It would be relevant to assess the size of the placebo effect on post-training performance.

In conclusion, six sessions of RSH at a simulated altitude of 1,500 m were effective to improve anaerobic performance during RSA and Wingate tests, and higher altitudes (at least until 3,200 m) did not provide further advantage. Most performance improvements consecutive to RSH were not correlated to individual pulse oxygen desaturation. However, analysis of HRV suggests that high altitude might induce greater disturbance of the autonomic nervous system activity raising the issue of a suitable period of time in evaluating performance after such a training program.

## Data Availability

The raw data supporting the conclusions of this article will be made available by the authors, without undue reservation.

## Abbreviations

BM, Body Mass; ES, Effect Size; FiO_2_, Fraction of Inspired Oxygen; HF, High Frequency; HR, Heart Rate; HRR, Heart Rate Recovery; HRV, Heart Rate Variability; LF, Low Frequency; RMSSD, Root Mean Square of Successive Differences; RPE, Rate of Perceived Exertion; RSA, Repeated-Sprint Ability; RSH, Repeated-Sprint training in Hypoxia; SpO_2_, Pulse Oxygen Saturation.
